# Mesenchymal stem cells suppress NF-κB and ERK signalling while enhancing chemotaxis in CD4^+^ T cells

**DOI:** 10.1038/s41598-025-14373-6

**Published:** 2025-08-30

**Authors:** Ezgi Sengun, Janeri Fröberg, Xuehui He, Marc Eleveld, Ruben L. Smeets, Hans J. P. M. Koenen, Katja Möller-Hackbarth, Tim G. A. M. Wolfs, Daan R. M. G. Ophelders, Martijn A. Huynen, Marien I. de Jonge, Renate G. van der Molen

**Affiliations:** 1 Department of Laboratory Medicine, Laboratory of Medical Immunology, Radboud University Medical Centre Nijmegen, Radboud Institute for Molecular Life Sciences, Route 469, Geert Grooteplein 10, PO-Box 9101, 6500 HB Nijmegen, The Netherlands; 2 Global Rare Disease, Chiesi Pharma AB, 17165 Solna, Sweden; 3 Department of Paediatrics, MosaKids Children’s Hospital, Maastricht University Medical Centre+, Maastricht, The Netherlands; 4 GROW, School for Oncology and Reproduction, Maastricht University, Maastricht, The Netherlands; 5 Center for Molecular and Biomolecular Informatics, Radboud University Medical Centre Nijmegen, Radboud University for Molecular Life Sciences, Nijmegen, 6525 GA The Netherlands

**Keywords:** CD4 ^+^ T, UC-MSCs, NF-κB, ERK, Immunomodulation, Migration, Signalling, Memory, Immunology, Stem cells

## Abstract

Inflammation is regulated by immune cells, with CD4^+^ T cells playing a key role in its progression and resolution. Modulating their response is crucial for controlling inflammation, and mesenchymal stem cells (MSCs) have emerged as a promising therapeutic target due to their immunomodulatory properties. We previously showed that umbilical cord derived MSCs (UC-MSCs) induce a memory response in TCR-activated CD4^+^ T cells, and here, we investigated the underlying mechanisms through gene expression analysis at different time points. Our results demonstrated that TCR activation is required for UC-MSCs to induce this memory response. Pathway analysis revealed that UC-MSCs induced the expression of genes that negatively regulate immune signalling pathways. This was further supported by phosphoflow cytometry, which showed suppression of the NF-κB and ERK pathways. Additionally, UC-MSCs enhanced the expression of genes related to CD4^+^ T cell adhesion and migration at 12 and 24 h. Notably, TNIP1 emerged as a potential key regulator of UC-MSCs-mediated immune modulation. This study provides new insights into how UC-MSCs influence CD4^+^ T cell responses and highlights molecular targets for further investigation into UC-MSCs-driven immune regulation.

## Introduction

T lymphocytes (T cells) are crucial components of the adaptive immune system, playing a key role in mediating cell-based immune responses to maintain homeostasis and protect against a range of diseases^1^.

During inflammation, naive T cells are activated and differentiate into effector cells following recognition of antigens and exposure to cytokines. After elimination of the antigens, a subset of T cells remains present as memory cells, possessing migratory and functional capabilities that enable a rapid response upon re-encounter with the antigen^2^.

The rapid changes in T cell function and phenotype during inflammation are regulated by several inflammatory pathways including the nuclear factor kappa-B (NF-κB) pathway, mitogen-activated protein kinase (MAPK) pathway, and Janus kinase/signal transducer and activator of transcription (JAK-STAT) pathway. The NF-κB pathway involved in pro-inflammatory processes including activation, proliferation and regulation of various cytokines such as IL-2, IL-6, and TNF- α^3^. The MAPK pathway, which comprises ERK, JNK, and p38 cascades, responds to signals from the T cell receptor (TCR) and cytokine receptors, leading to T cell activation and differentiation into subsets such as Th1, Th2, and Th17^4^. The JAK-STAT pathway is triggered by the binding of cytokines to their respective receptors, resulting in the activation of genes involved in T cell differentiation and cytokine production^5^. Collectively, immune signalling pathways orchestrate the activation, proliferation, differentiation, and effector functions of CD4^+^ T cells, thereby shaping the immune response during inflammation.

Dysregulated inflammation can lead to chronicity, associated with an intense and prolonged infiltration and activation of various immune cells^6,7^. In this respect, mesenchymal stem cells (MSCs) have emerged as a promising tool to resolve inflammation due to their immunomodulatory capacity and regenerative potential^8^. While bone marrow derived MSCs were traditionally the most commonly used for clinical interventions and research, umbilical cord derived MSC (UC-MSCs) are gaining attention due to their primitive nature, higher proliferative capacity, lower immunogenicity due to minimal expression of MHCI/II^9^.

MSCs were shown to suppress proliferation of CD4^+^ T cell subsets ^10,11^. Moreover, MSCs facilitate the formation of regulatory T cells (Treg, CD4^+^CD25^+^FOXP3^+^) from conventional T cells^12,13^, and promote a shift from pro-inflammatory Th1 to anti-inflammatory Th2 cells including a change in the cytokine profile towards anti-inflammation^14,15^. Additionally, it has been shown that pro-inflammatory Th17 cells adhere to MSCs leading to induction of regulatory phenotype in Th17 cells^16^. Emerging evidence suggests that MSCs-derived extracellular vesicles (MSC-EVs) play a crucial role in immunomodulation by influencing T cell differentiation and function^17^. These MSC-EVs contribute to the suppression of T cell proliferation, alteration in cytokine production and promoting Treg formation^18,19^. While there are numerous studies showing the effects of MSCs and MSC-EVs on T cell differentiation and function in inflammation, the underlying changes in the signalling pathways have not been fully elucidated.

In our previous study, we investigated the immunomodulatory capacity of UC-MSCs on CD4 ^+^ T cells. We demonstrated that UC-MSCs induce a memory response in TCR-stimulated CD4^+^ T cells in a time-dependent manner^7^. In this study, we are investigating the underlying mechanism and molecular targets which UC-MSCs modulate to induce memory response. We performed RNA sequencing on TCR-activated CD4 ^+^ T cells co-cultured with UC-MSCs and compared their transcriptomic profiles to TCR-activated CD4 ^+^ T cells cultured without UC-MSCs.

Our findings indicate that UC-MSCs are key modulators of inflammatory function and T cell migration, primarily through the alteration of signal transduction pathways. This analysis highlights several promising gene candidates for further investigation.

## Methods

### Ethical statements

The ethical approval for using healthy blood controls was obtained from the Erasmus MC Medical Ethics Committee (METC), under project number NL40331.078. The study titled “Primary Immunodeficiencies: Immunological and Genetic Background in Relation to Clinical Complications” was granted ethical consent on January 19, 2021. Prior to blood sampling, donors signed a written informed consent for scientific use according to Dutch law.

For UC-MSCs products, ethical approval for the project titled “Chiesi AMSTEM H2020 Grant Application” was obtained from the Institutional Review Board (IRB) overseeing the donor’s informed consent process at Lonza Walkersville, Inc. The approval ensures compliance with the ethical standards for the procurement of human umbilical cord tissue for research use only (ROU). The tissue was procured under the regulations specified by the IRB, and the donor provided informed consent for research purposes only without receiving compensation.

All methods were performed in accordance with the relevant guidelines and regulations.

### Peripheral blood mononuclear cell isolation and CD4^+^ T cell purification

Blood samples for peripheral blood mononuclear cells (PBMC) were collected in 10 mL EDTA tubes (BD, Labware, NJ). PBMC were isolated using AutoMACS (Miltenyi, Biotec, GmbH, Bergisch Gladbach, Germany) according to manufacturer’s guidelines. CD4^+^ T cells were isolated by positive selection using CD4^+^ T cell isolation kit (Miltenyi) with AutoMACS according to manufacturer’s instructions. CD4^+^ T cells were resuspended in complete RPMI 1640 medium (Life technologies), supplemented with 10% human pooled serum (HPS, manufactured in house), 1 mM sodium pyruvate, 2 mM glutamax, 100 U/mL penicillin, and 100 ug/mL streptomycin (all Thermo Fisher Scientific). The purity of the sorted CD4^+^ T cells was above 95%.

### Umbilical cord mesenchymal stem cells (UC-MSCs) culturing

UC-MSCs were provided by Chiesi Farmaceutici S.p.A, Italy at passage 4 and used without further passaging. UC-MSCs were cultured in complete growth medium which contains 2000 U/L heparin (Leo Pharma B.V., Copenhagen, Denmark) and 5% human platelet lysate (provided by Chiesi Farmaceutici S.p.A, Italy).

UC-MSCs were thawed at 37 º C for 4–6 min. UC-MSCs were centrifuged at 500 × *g* for 10 min at room temperature and the pellet was dissolved in 15 mL complete UC-MSCs growth medium. After trypan blue counting, cells were seeded at a density of 1.5 × 10^4^ viable cells per cm^2^ (1.1 × 10^6^/flask) in T75 flasks for recovery. The flasks were placed in 37 °C, 5% CO_2_, 95% humidity incubator for 48 h. After the recovery, UC-MSCs medium was aspirated, and cells were washed once with sterile PBS (Fresenius Kabi, Bad Homburg, Germany). UC-MSCs were harvested by using 3U/mL trypsin/EDTA (Gibco®Thermo Fisher Scientific, Waltham, USA). Trypsinization was neutralized by using 5 ml cold basal UC-MSCs culture medium without platelet lysate. Cells centrifuged at 300 × *g* for 10 min at 4 °C. Cells were washed after aspirating the medium and counted with trypan blue for further usage.

The phenotyping of UC-MSCs was previously performed by our group as part of another study and confirmed that the cells were negative for CD14, CD19, HLA-DR, and CD45. They were positive for the stem cell markers CD73, CD90, and CD105^20^, consistent with findings reported in other studies as well^21^.

### UC-MSCs and CD4^+^ T cell co-culture

Freshly isolated 1.5 × 10^6^ CD4^+^ T cells were seeded in 12 well plate with complete RPMI medium and were activated with αCD3/CD28 beads (Dynabeads™ Human T-Activator, Gibco®Thermo Fisher Scientific) in 1:5 (bead-to-cell) ratio. After 24 h of pre-stimulation with αCD3/CD28 beads, CD4^+^ T cells were co-cultured with UC-MSCs in a 1:5 (UC-MSCs:CD4^+^ T) cell ratio as previously described^7^. A previous analysis with two UC-MSCs:PBMC ratios (1:2.5 and 1:5), along with different bead-to-cell ratios (1:5 and 1:10), revealed more pronounced effects on the percentages of activated CD4^+^ (Ki67^+^ and CD25^+^) and activated CD8^+^ (Ki67^+^) cells at an UC-MSCs:PBMC ratio of 1:5 and a bead-to-cell ratio of 1:5 (data not shown). Therefore, subsequent experiments were conducted using the 1:5 UC-MSCs:CD4^+^ ratio and 1:5 bead:cell ratio.

Total RNA was isolated from 3 experimental condition (stimulated T cells, unstimulated T with UC-MSCs and stimulated T with UC-MSCs) after 2 h,12 h and 24 h of co-culture. 3 different biological donors were used.

### RNA isolation and sequencing

Total RNA was isolated using RNeasy minikit (QIAGEN, Hilden, Germany) according to manufacturer’s instructions. Isolated products were eluted in nuclease free molecular grade water. Quality of RNA was immediately determined by using Agilent RNA 6000 Nano kit on an Agilent 2100 Bioanalyzer system (Agilent Technologies, Palo Alto, CA). Samples that have RNA Integrity Number (RIN) > 8.0 and concentration > 2 ug/total RNA were sent to BaseClear B.V. (Leiden, The Netherlands) for library preparation and Illumina sequencing. FASTQ files were obtained from the company for further analysis.

### Quality validation and read alignment

Quality of sequence read in 27 FASTQ files was evaluated using MultiQC analysis, including mean (Supplemental Fig. 1A) and per sequence (Supplemental Fig. 1B) quality scores, and per sequence GC content (Supplemental Fig. 1C). All 27 samples were of high quality and suitable for downstream bioinformatic analysis. Sequence reads were aligned to human Homo_sapiens.GRCh38.99 reference transcriptome using STAR aligner version 2.7.11a^22^. Per sample, a total of 18,956,888 reads were processed, of which 15,914,523 (84.0%) were successfully mapped to genomic features and counted.

### Data transformation and downstream analysis

The transcript alignment data stored in BAM files, were fed into *featureCounts* for obtaining the count matrix. Specific information about the samples were kept in an information sheet. A DeSeq object was created from count matrix and information sheet using *DESeqDataSetFromMatrix* function in R. The data were processed and normalized using *DESeq2* function and differentially expressed genes (DEG) were detected by calculating statistics and p-values using the *results* function. Screened DEG were mapped to Gene Ontology term (GO), among which biological process was used. GO—Biological Process analysis involves categorizing genes based on the biological processes they influence, such as cell division or immune response. This analysis helps identify which processes are overrepresented among differentially expressed genes, providing insights into the biological impact of these changes^23^. All the figures were made by using R software (version 4.2.2).

### CIBERSORT: immune cell landscape

The CIBERSORT algorithm was used to estimate relative proportions of different immune cell types^24^. This algorithm uses a signature matrix which contains the pre-defined gene expression matrix to infer the abundance of immune cell populations. As an input file, transcripts per million (TPM) values were calculated from the count matrix. To minimize the variations in sequencing depth and length, TPM values were normalized, and gene lengths were retrieved from GENCODE v38 annotation file. The TPM values were then used as input for CIBERSORT analysis.

### Flow cytometry measurement

For flow cytometry analysis, 1.0 × 10^5^ CD4^+^ T cells were initially stimulated with αCD3/CD28 beads at a 1:5 bead-to-cell ratio for 24 h^7^. The following day, the activated CD4^+^ T cells were co-cultured with UC-MSCs at a 1:5 UC-MSCs-to-CD4^+^ T cell ratio in a 96-well plate. After 24 h of co-culture, cells were stained with monoclonal antibodies targeting surface markers (Table 1) for 30 min on ice. Subsequently, the cells were fixated with fixation buffer (BD Cytofix) for 10 min in the incubator 37ºC, followed by washing with FACS buffer (0.2% bovine serum albumin in PBS, Fresenius Kabi). Next, the cells were permeabilized with perm buffer IV (BD Phosflow) on ice for 20 min and washed again with FACS buffer. Intracellular markers (Table 1) were added for 30 min and kept on ice. After two additional washes with FACS buffer, cells were resuspended in 100 µL of FACS buffer and analysed using the Sony ID7000™ Spectral Analyzer. The viability of the CD4^+^ T cells were above 90% throughout the culture. Flow cytometry data was analysed using Kaluza software. All the plots were made by using R software (version 4.2.2).

**Table 1:** List of monoclonal antibodies used in flow cytometry.

Antibody	Company	Catalogue Number	Staining
CD3- SparkBlu550	BioLegend	344852	Surface
CD4-FITC	Dako	F0766	Surface
CD25-BV605	BioLegend	356142	Surface
CD45-SparkBlu574	BioLegend	368558	Surface
CD45RA-Pacific Blue	BioLegend	304118	Surface
CD197-BV421	BioLegend	353208	Surface
Zombie-NIR	BioLegend	423106	Surface
Ki67-AF647	BD Bioscience	558615	Intracellular
NFkB- PE CF594	BD Bioscience	565447	Intracellular
ERK- PE Cy5	BioLegend	369513	Intracellular
P38MAPK – eFluor710	Invitrogen	46-9078-41	Intracellular

## Results

### UC-MSCs-induced memory formation and reduced the activation status of CD4^+^ T cells

In our previous study, we investigated the prolonged effects of UC-MSCs on pre-stimulated CD4^+^ T cells by assessing phenotypic changes after 24 h, 72 h, and 96 h of co-culture. UC-MSCs-induced alterations in surface protein expression on CD4^+^ T cells were detectable as early as 24 h, and by 72 h, UC-MSCs had promoted a memory phenotype. We also demonstrated that within the first 24 h, UC-MSCs-derived paracrine factors alone were sufficient to reduce activation and proliferation in CD4^+^ T cells. However, the development of a memory phenotype required both mechanisms^7^.

Based on these previous findings, in this study, we aimed to uncover the molecular mechanisms underlying UC-MSCs-induced memory formation in CD4^+^ T cells. Since phenotypic effects, based on the expression of cell-surface markers, became detectable after 24 h of co-culture, we performed RNA sequencing on CD4^+^ T cells at 2 h, 12 h, and 24 h of co-culture, as transcriptional changes precede protein-level modifications.

**Fig. 1:**
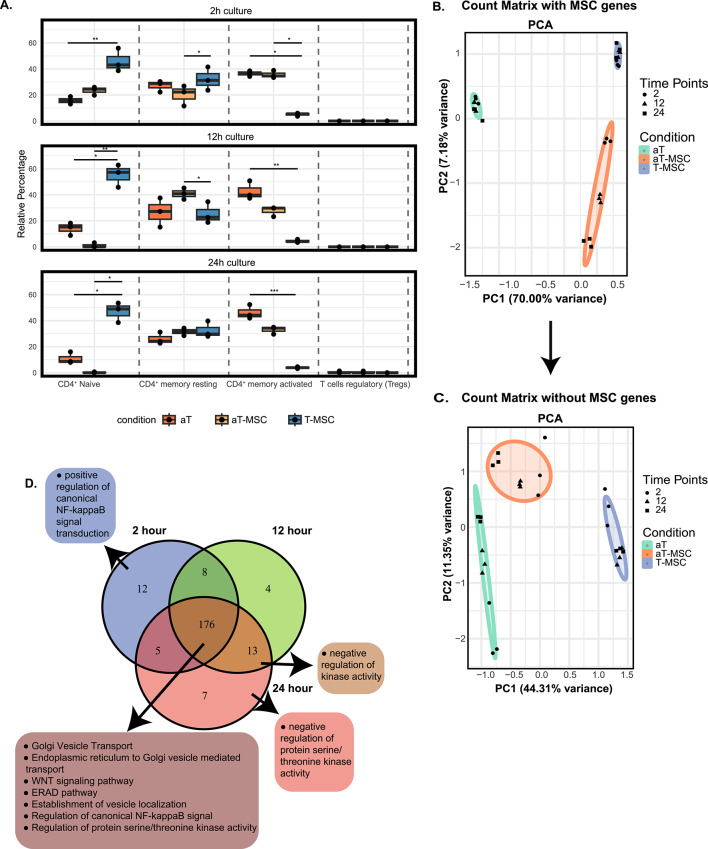
UC-MSCs induce a memory response on CD4^+ ^T cells in a time dependent manner. (**A**) Immune landscape differences between stimulated T cells (aT), stimulated T cells co-cultured with mesenchymal stem cells (aT-MSC), and T cells co-cultured with mesenchymal stem cells (T-MSC) were analysed over 2 h, 12 h, and 24 h culture periods by using CIBERSORT. Four distinct cell populations were annotated on the x-axis, with their relative percentages displayed on the y-axis. Statistical comparison was performed using Dunn’s Multiple Comparison test. *p < 0.05, **p < 0.01, ***p < 0.001. (**B**) PCA analysis was performed to show variability between biological donors, time and conditions. The difference between UC-MSCs containing conditions (aT-MSC and T-MSC) and aT suggest the variance is caused by UC-MSCs genes in the PC1 axis. (**C**) *loadings* function was used to find UC-MSCs-specific genes that cause the variance in PC1 axis. These genes were filtered out from the count matrix to obtain UC-MSCs-free counting matrix. The filtered genes then used as representative of UC-MSCs-specific genes. Gene Ontology Biological Processes (GO-BP) was performed in each time point. (**D**) Venn-diagram was made to show similarity and differences between each time point in aT-MSC condition based on GO-BP analysis. 3 different biological donors were used.

The immune cell composition based on RNA level was assessed by CIBERSORT analysis. CIBERSORT was developed to address the challenge of quantifying the contribution of individual cell types to bulk gene expression profiles. Using this tool, we identified various CD4⁺ T cell–related phenotypes as the predominant immune cell components in the MSC co-culture system^24,25^ As expected, the CD4^+^ T cell related phenotypes were the primary immune cell components in the co-culture with UC-MSCs (Fig. 1A). Co-cultures of unstimulated T cells (T) with UC-MSCs (T-MSC) contained the highest percentage of naïve T cell compared to other conditions. Similarly to what we found previously, the memory cell phenotype was elevated in TCR activated CD4^+^ T cells (aT) with UC-MSCs co-cultures (aT-MSC) relative to aT conditions alone, as described previously^7^. Among the two memory phenotypes, activated memory T cells (CD45RO^+^CD69^+^CD25^+^^24^) and resting memory T cells (CD45RO^+^CD62L^+^CD69^-^CD25^-^^24^), activated memory T cells were downregulated in the aT-MSC, suggesting that UC-MSCs impair the activation status while maintaining the memory phenotype. To further support this concept, flow cytometry analysis was performed on the co-cultures, observing a trend toward reduced activation and proliferation in aT-MSC conditions compared to aT alone (Supplementary Fig. 2A). Additionally, there was a decrease in the effector memory population (Supplementary Fig. 2B) and an increase in the central memory response (Supplementary Fig. 2C).

Principal component analysis (PCA) was performed to determine the variation in donors, sample group and time points. As expected, the PCA plot (Fig. 1B) revealed a significant separation of UC-MSCs-containing conditions (aT-MSC and T-MSC) from the aT condition along the PC1 axis, reflecting the contribution of the UC-MSCs to the RNA data. The dominant effect of the UC-MSCs to the overall gene expression masks the effect of the UC-MSCs on the T cell specific genes. To filter out the effect of the UC-MSCs on the overall gene expression, we removed the genes whose expression was positively correlated with the PC1 axis (15708 genes) from the count matrix (37479 genes in total). Removing the genes from the count matrix reduced the variance on the PC1 axis from 70.00% to 44.31% (Fig. 1C), resulting in closer clustering of aT and aT-MSC, which provided a more focused view of the T cell gene profile. The removed genes were stored and will be referred to as UC-MSCs-related genes. These genes allow us to focus on the effects of co-culture specifically on UC-MSCs gene expression.

Notably, the removal of UC-MSCs-related genes eliminated the observed time difference in the aT-MSC condition, highlights the potential time-dependent changes in UC-MSCs- related gene expression within aT-MSC. Gene ontology (GO) analysis of biological processes (GO-BP) was performed on UC-MSCs-related genes from different time points (Fig. 1D, Supplementary Data 1). Across all time points, genes associated with NF-κB, ERAD, and WNT signalling pathways were identified. More strikingly, genes involved in MSC-EVs formation, transport, and localization were also found, suggesting a potential mechanism of action on T cells regardless of TCR activation. Comparison of UC-MSCs-related pathways between aT-MSC and T-MSC conditions (Supplementary Data 1) were done by using setdiff() function from R, but no differences were observed.

### Stimulation is necessary to generate a memory response at 24 h of co-culture

**Fig. 2:**
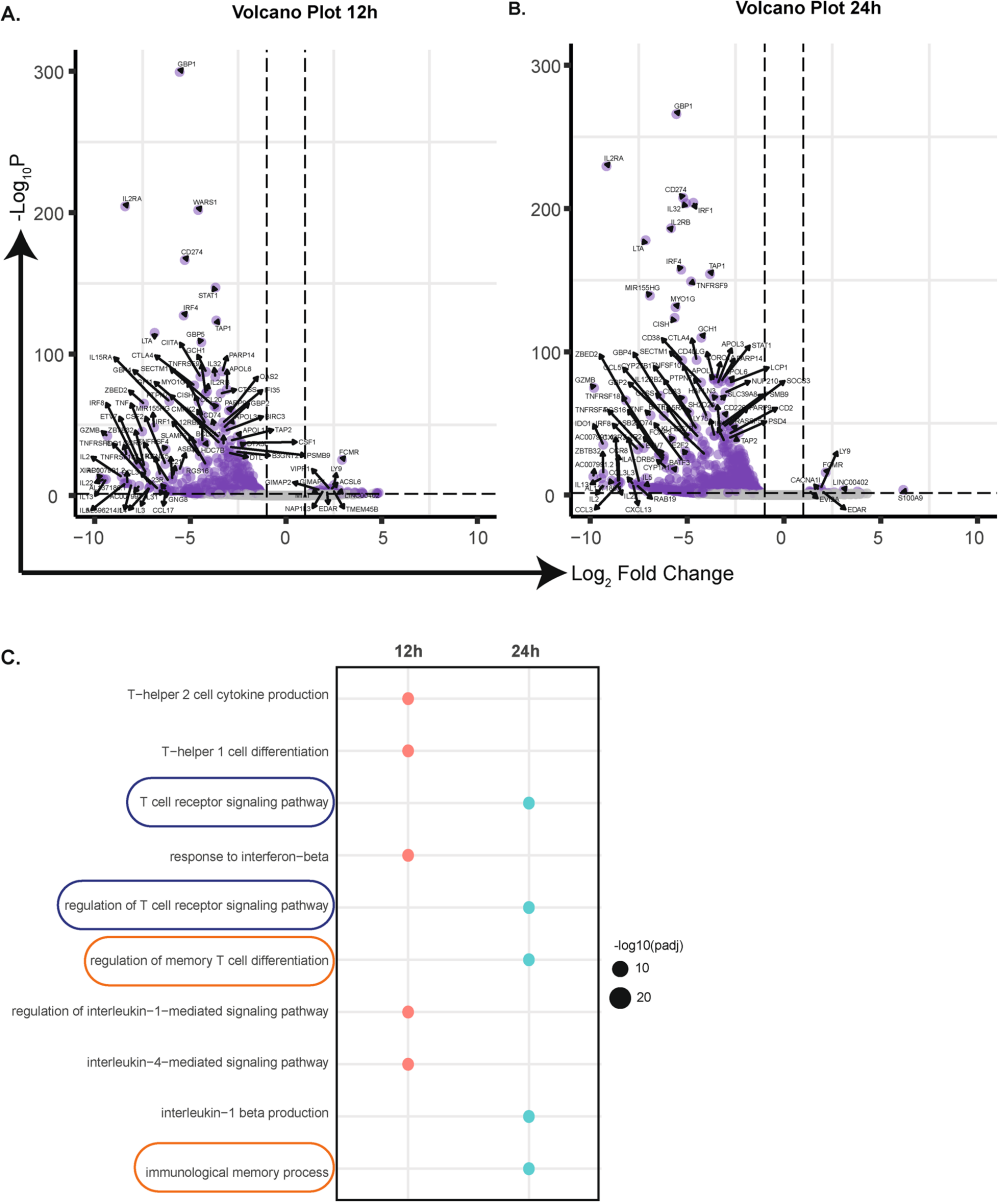
TCR-specific stimulation is necessary for memory induction. (**A**,**B**) Volcano plot representing significantly differentially expressed genes between in T-MSC over baseline aT-MSC at 12 h and 24 h with respect to -log10p in the y-axis and log2 fold change in the x-axis. (**C**) All significant genes were selected from both time points and GO-BP was performed and unique pathways per time point was presented.

In the new PCA plot without UC-MSCs genes (Fig. 1C), three clusters were formed, representing different experimental conditions. There was a clear difference between stimulated (aT-MSC) and non-stimulated (T-MSC) samples, highlighting the impact of stimulation. Since a more distinct separation in the abundance of resting memory and activated memory phenotypes was observed at 12 h and 24 h between aT-MSC and T-MSC, we focused on the differential gene expression of aT-MSC relative to baseline T-MSC at 12 h (Fig. 2A) and 24 h (Fig. 2B).

Genes that significantly differ in expression between two condition and have an absolute log2 fold change value greater than 1, were filtered. GO-BP was performed on both mutually (Supplementary Data 2) and distinctively (Fig. 2C) expressed genes among the time points 12 h and 24 h. Strikingly, among many pathways(Supplementary Data 2), T cell receptor signalling and memory T cell related pathways were more pronounced in aT-MSC at 24 h compared to 12 h.

### UC-MSCs induced chemotaxis related genes in TCR activated CD4^+^T cells

**Fig. 3:**
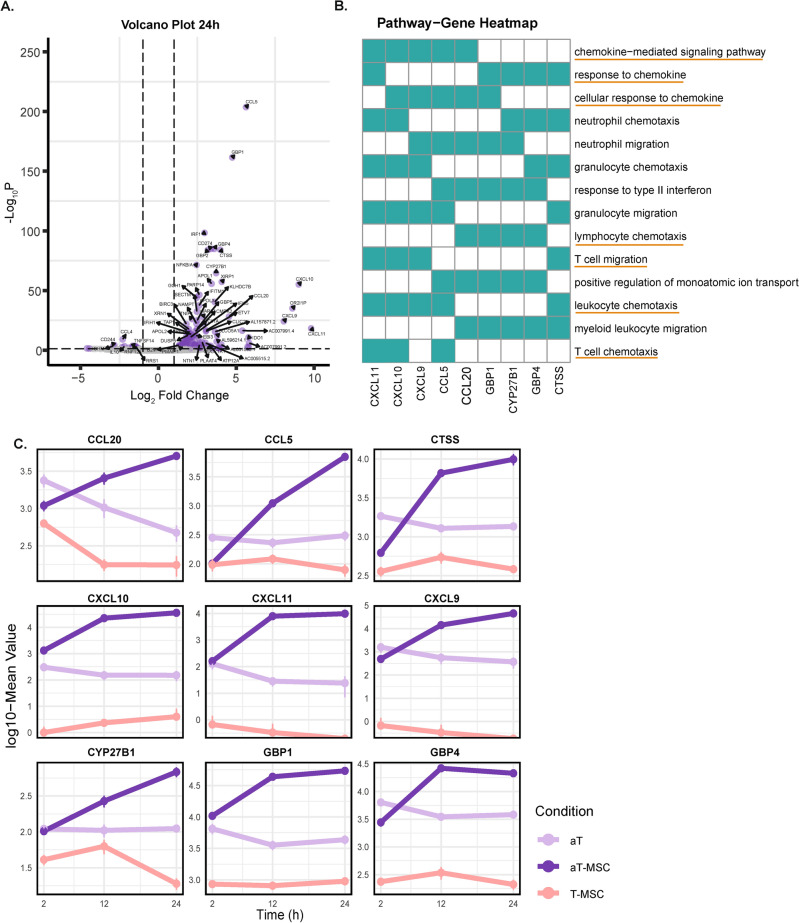
UC-MSCs induce chemotaxis genes. (**A**) Volcano plot depicting differentially expressed genes in aT-MSC compared to aT at 24 h. (**B**) Upregulated genes were ranked based on the highest log2 fold change and significance. The top 15 ranked genes were compared to the CIBERSORT signature matrix to identify those correlated with memory response. Among these, 9 genes were further analysed through GO-BP to determine their associated biological processes. (**C**) Line graph illustrating the dynamic changes in gene expression over time between the conditions. Each time point represents the mean value derived from three different biological donors.

On basis of the PCA plot without UC-MSCs genes (Fig. 1C) we next focussed on the difference between aT and aT-MSC clusters, which highlights the effect of UC-MSCs stimulation. Since Fig. 2 shows that memory-related genes were upregulated after 24 h of stimulation, we generated a volcano plot to identify UC-MSCs-regulated genes linked to the memory response by comparing aT to aT-MSC. We could identify 90 genes that were upregulated in the aT-MSC compared to the aT (Fig. 3A). These genes were ranked based on having the highest fold2 change and being significant. The top 15 significant genes were selected and compared to the CIBERSORT signature matrix, enabling the identification of genes associated with the memory response. Of these 15 genes, 9 were found to correspond to memory response based on the CIBERSORT signature matrix. These 9 genes were used for GO-BP analysis. CXCL11, CXCL10, CXCL9, CCL5, CCL20, GBP1, CYP27B1, GBP4 and CTSS were mainly correlated with chemokine response and lymphocyte chemotaxis (Fig. 3B). To observe the dynamic regulation of these genes, we monitored the expression level over time (Fig. 3C). The top 9 genes were significantly (Supplementary Fig. 2) upregulated at 12 h in aT-MSC compared to aT.

### NF-κB and ERK pathways are hallmark for immunomodulatory function of UC-MSCs

**Fig. 4:**
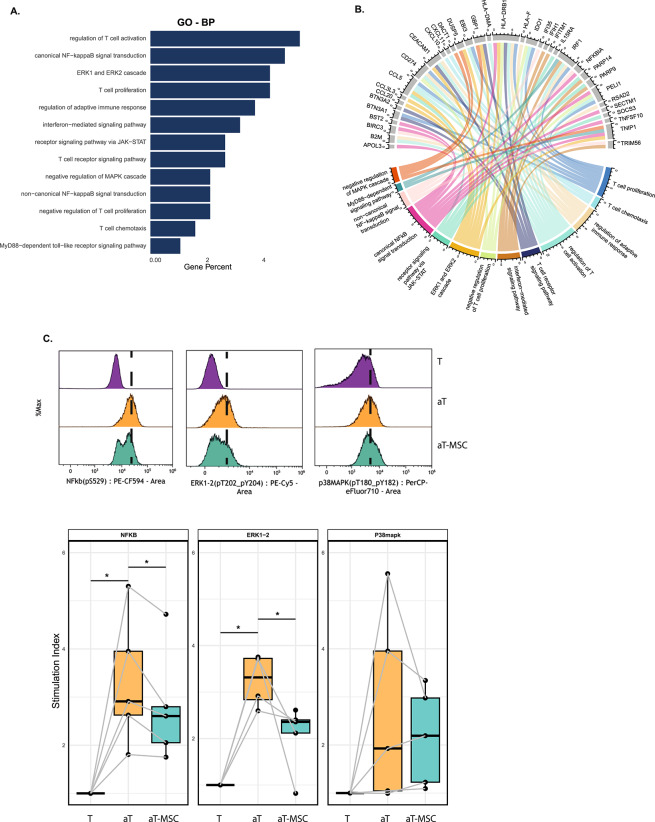
NF-κB and ERK pathways are downregulated by UC-MSCs. (**A**) GO-BP analysis was performed on genes upregulated by UC-MSCs, as identified in the volcano plot. Pathways related to immune signalling were selected for further examination. (**B**) Chord diagram illustrating each gene contributing to the identified signalling pathways. (**C**) The upper panel shows representative plots for the mean fluorescence intensity (MFI) of each pathway marker, comparing conditions: unstimulated CD4^+^ T cells (T), stimulated CD4^+^ T cells (aT), and stimulated CD4^+^ T cells with UC-MSCs (aT-MSC). The lower panel includes boxplots summarizing the data from five biologically different donors. The phosphorylation of p65 (PE-CF594, NF-κB pathway), ERK1/2 (PE-Cy5, ERK pathway), and p38 (eFluor710, MAPK pathway) was measured across T, aT, and aT-MSC conditions. Samples were measured using Sony ID7000™ and analysed in Kaluza Software. Statistical comparisons were performed using the paired-t test. *p < 0.05, **p < 0.01, ***p < 0.001.

To better understand the modulatory mechanism of UC-MSCs, GO-BP analysis was performed on all genes that were differentially expressed with a log2 fold change higher than 1 in aT-MSC compared to aT at 24 h (Supplementary Fig. 4). Among the listed top 15 pathways, the *immune response-regulating signalling pathways* were the most remarkable one, since stimulated T cells can activate signalling pathways through TCR engagement and/or paracrine factors. For instance NF-κB, MAPK, ERK, and JAK-STAT pathways are crucial for regulating T cell responses. In addition, MyD88 serves as an upstream regulator in these signalling cascades, initiating activation that subsequently involves NF-κB, MAPK, and JAK-STAT pathways. NF-κB can also be independently activated to influence T cell functions. The ERK cascade, specifically, is a downstream component of the MAPK pathway, contributing to T cell proliferation and differentiation^26^. Subsequently, we further went through the list of all signalling pathways (Supplementary Data 3) and selected the specific pathways mentioned above (Fig. 4A). To gain functional insights into UC-MSCs activity and hypothesize about key target molecules, we identified genes associated with each pathway (Fig. 4B). In the MAPK pathway, the TNIP1 and DUSP5 genes, both associated with the downregulation of the ERK pathway^27,28^, were upregulated in the presence of UC-MSCs. Similarly, in the NF-κB pathway, the inhibitory genes TNIP1 and NFKBIA were upregulated^29^. Additionally, the NF-κB pathway related genes, SECTM1 and BST2, were upregulated in the co-cultures which are related to cell adhesion and migration. In the MAPK/ERK pathway, genes such as CEACAM1, CCL5, CCL3L3, and CCL20, responsible for cell–cell adhesion and migration, were also upregulated. Furthermore, anti-apoptotic genes TRIM56 and BIRC3 showed increased expression in the presence of UC-MSCs, suggesting enhanced cell survival.

After identifying immune signalling pathways and their associated genes through RNA sequencing, we further analysed the activation levels of these pathways at the protein phosphorylation level. Specifically, we quantified the phosphorylation of key proteins, including p65 from the NF-κB pathway and ERK1/2 and p38 from the MAPK pathway, to gain insights into the dynamics of immune signalling. Comparing T and aT conditions, we observed an increase in the phosphorylation levels of NF-κB, ERK1-2, and p38 proteins (Fig. 4C, upper panel). However, in aT-MSC conditions, there was a significant reduction in the phosphorylation of NF-κB and ERK1-2, but not p38 (Fig. 4C). T-MSC did not differ from T alone; therefore, it was not included in the graphs. Overall, NF-κB and ERK, but not p38 pathway were found to be downregulated by UC-MSCs at both RNA and protein level.

## Discussion

Earlier studies have demonstrated that UC-MSCs can regulate the T cell response by shifting their phenotype from pro-inflammatory Th1 to anti-inflammatory Th2 or Treg^30–32^. We previously showed that UC-MSCs induced a memory response in CD4^+^ T cells starting from 72 h^7^. As a follow-up study, we aimed to investigate the mechanisms underlying the UC-MSCs-induced memory response in CD4^+^ T cells and to explore the broader implications of UC-MSCs interactions in immune regulation by performing RNA sequencing.

CIBERSORT analysis of aT, aT- MSC, and T- MSC conditions at different time points revealed a decrease in the activated memory phenotype and an increase in the resting memory phenotype in aT- MSC compared to aT, starting at 12 h. Differential gene expression analysis showed upregulation of memory-specific genes at 24 h. However, flow cytometry measurements did not show a significant difference in the memory response between aT and aT-MSC conditions at 24 h (Supplementary Fig. 2). This suggests that 24 h may be too short to detect changes of surface markers, but a trend indicates potential alterations at later time points. In the absence of T cell stimulation (T-MSC), naïve T cells were predominantly enriched at the RNA level compared to aT-MSC. Other studies have shown that MSCs can suppress Th1 differentiation specifically in specifically activated cells^33^. Furthermore, research indicates that MSCs inhibit activation and enhance cell viability more prominently under stimulated conditions compared to without stimulation^7,34^. These studies suggest that the activation state plays a crucial role in how UC-MSCs modulate the immune response.

Analysis of UC-MSCs specific genes showed constant activation of MSC-EVs formation pathway in both aT-MSC and T-MSC conditions. The findings described in this study are consistent with our previous publication, which demonstrated that within 24 h, the paracrine factors were as effective as direct UC-MSCs-T cell contact in modulating the immune response. However, for sustained long-term effects, direct cell contact was found to have a more significant impact^7^.

Analysis of the top 9 genes showing increased expression in aT-MSC at 24 h, which correlated with the memory response, revealed significant involvement in chemotaxis and migration. The genes encoding for chemokines CXCL9, CXCL10 and CXCL11, known to be involved in the recruitment of immune cells to inflammation sites, were highly expressed ^35–37^. These chemokines are primarily expressed by Th1 and memory T cells. Enhanced T cell migration is vital for a quick response to injury, caused by for example pathogen invasion, preventing severe infection and facilitating tissue repair^35,36^. The targeted recruitment of memory T cells improves immune response efficiency, potentially reducing collateral damage^38^. The ability of UC-MSCs to enhance T cell migration suggests their therapeutic potential in inflammatory diseases, by improving the precision and localization of immune cell responses in therapies^39,40^.

Analysis of the genes that were upregulated by UC-MSCs, revealed that many of these genes are involved in the negative regulation of various signalling pathways. Among the most noteworthy are TNIP1, DUSP5, and NFKBIA. TNIP1 is a critical negative regulator of the NF-κB pathway. It interacts with NEMO (NF-κB Essential Modulator), a key component in the NF-κB signalling cascade, also known as IKKγ which is a regulatory subunit of the IKK complex. This interaction inhibits the IKK complex activation, thereby preventing the phosphorylation and subsequent degradation of IκB proteins. As a result, NF-κB dimers remain sequestered in the cytoplasm preventing their translocation to the nucleus and the transcription of pro-inflammatory genes^41,42^. Moreover, TNIP1 acts as an adaptor protein for A20, a ubiquitin-editing enzyme that has a key role in terminating NF-κB signalling. By facilitating the interaction between A20 and its substrates, TNIP1 enhances A20’s ability to remove ubiquitin chains from signalling molecules, thereby, inhibiting NF-κB activation and dampening inflammatory response^43^. Beyond the NF-κB pathway, TNIP1 also targets upstream elements of the MyD88-dependent signalling cascade, such as IRAK1 and TRAF6, to suppress inflammatory responses^28^. This suppression results in reduced secretion of NF-κB-mediated cytokines, including TNF-α, IL-6, and IL-1β^44^. Genetic studies have linked TNIP1 variants to conditions such as systemic lupus erythematosus (SLE)^45^, psoriasis^46^, and rheumatoid arthritis (RA)^47^. By controlling these critical signalling pathways, TNIP1 helps to maintain immune homeostasis and prevent chronic inflammation leading to autoimmune diseases. The multifaceted inhibition by TNIP1 highlights its vital role in controlling excessive inflammation and makes it a potential target for therapeutic interventions.

NFKBIA encodes an inhibitor of NF-κB pathway, which prevents NF-κB dimers from phosphorylating and translocating to the nucleus, thereby reducing NF-κB activity^48,49^. DUSP5 is a negative feedback regulator in the MAPK/ERK pathway, dephosphorylating ERK and downregulating ERK signalling. This action reduces proliferative signals and helps to maintain cellular homeostasis by preventing hyperactivation^27,50^.

Based on our RNA expression results, we observed the upregulation of NFKBIA, TNIP1, and DUSP5, which are key negative regulators of immune signalling pathways in the presence of UC-MSCs. To further assess the impact on pathway activation, we measured phosphorylation levels of pathway-specific proteins. For the MAPK pathway, we evaluated ERK1/2 and p38, and for the NF-κB pathway, we focused on p65. Notably, flow cytometry data revealed that UC-MSCs selectively downregulated the NF-κB and ERK signalling pathways, while p38 from the MAPK pathway remained unaffected.NF-κB is a key transcription factor involved in regulating inflammatory responses. Its downregulation likely reduces T cell activation and inflammatory cytokine production. ERK, part of the MAPK pathway, plays a role in cell growth, differentiation, and survival. The downregulation of ERK signalling suggests that UC-MSCs influence T cell proliferation, differentiation, or activation^51^. This aligns with our CIBERSORT analysis and previous findings, which show that UC-MSCs suppress CD4^+^ T cell activation by reducing CD25 expression. Interestingly, UC-MSCs did not affect p38 activation, which is involved in stress responses and inflammation. Notably, p38 also regulates NF-κB transcriptional activation in response to stimuli^52^. This suggests that while UC-MSCs selectively target NF-κB and ERK pathways to modulate inflammation, they may leave p38 signaling intact, potentially allowing CD4^+^ T cells to respond to other stimuli when necessary.

In addition to negative regulators, we identified upregulated genes (CEACAM1 and CCL3LC) associated with cell adhesion and migration. This further highlights the role of UC-MSCs in enhancing chemotaxis.

Our study revealed alterations in signalling cascades and identified several candidate proteins that may contribute to the regulation of pathways influenced by UC-MSCs, providing new insights into the molecular mechanisms underlying their immunomodulatory functions. These findings complement previous research demonstrating that MSCs can suppress T cell receptor (TCR) signalling by modulating the phosphorylation status of key signalling molecules, including CD3ζ, LCK, ZAP-70, and PLC-γ1^53^. Furthermore, other studies have implicated miRNA-125b and miRNA-155, both of which are regulated by MSC treatment, in the TCR signalling pathway, linking their expression to the suppression of CD4⁺ T cell activation^54^.

A limitation of this study is the absence of an UC-MSCs-only control group. Since our focus was on the overall biological effects of UC-MSCs on CD4^+^ T cells, we did not include an UC-MSCs-only condition. Although we lacked UC-MSCs-specific genes, we could identify UC-MSCs-related genes shared between T-MSC and aT-MSC conditions by comparing them with aT-only. However, this approach did not allow us to assess the if effect of UC-MSCs depends on the culture conditions.

## Conclusion

Overall, our findings demonstrate that UC-MSCs induce a resting memory response according to CIBERSORT and pathway analysis in TCR-activated CD4^+^ T cells by suppressing the activity of the NF-κB and ERK pathways. Additionally, UC-MSCs enhance the expression of genes related to migration and chemotaxis in CD4^+^ T cells. Notably, we identified a potential gene candidate, TNIP1, that may play a crucial role in how UC-MSCs modulate immune responses in CD4^+^ T cells. This study provides new insights into how UC-MSCs mechanistically influence the CD4^+^ T cell response and opens a new avenue for further functional investigation into the specific genes and proteins through which UC-MSCs modulate immune signaling pathways.

## Supplementary Information

Supplementary Information 1.

Supplementary Information 2.

Supplementary Information 3.

Supplementary Information 4.

## Data Availability

All sequencing and flow cytometry data supporting this study are available to other investigators without restrictions. The datasets generated and/or analysed during the current study are available in the Radboud Data Repository, PREMSTEM Collection, doi: 10.34973/cpn0-p348.
