# Supplementary material for: Mesenchymal stem cells suppress NF-κB and ERK signalling while enhancing chemotaxis in CD4+ T cells
Source: Sci Rep. 2025 Aug 30;15:32000. doi: 10.1038/s41598-025-14373-6 (PMC12398613; doi:10.1038/s41598-025-14373-6)
Supplement: Supplementary file 4 — Supplementary Information 4. [file 41598_2025_14373_MOESM4_ESM.pdf]

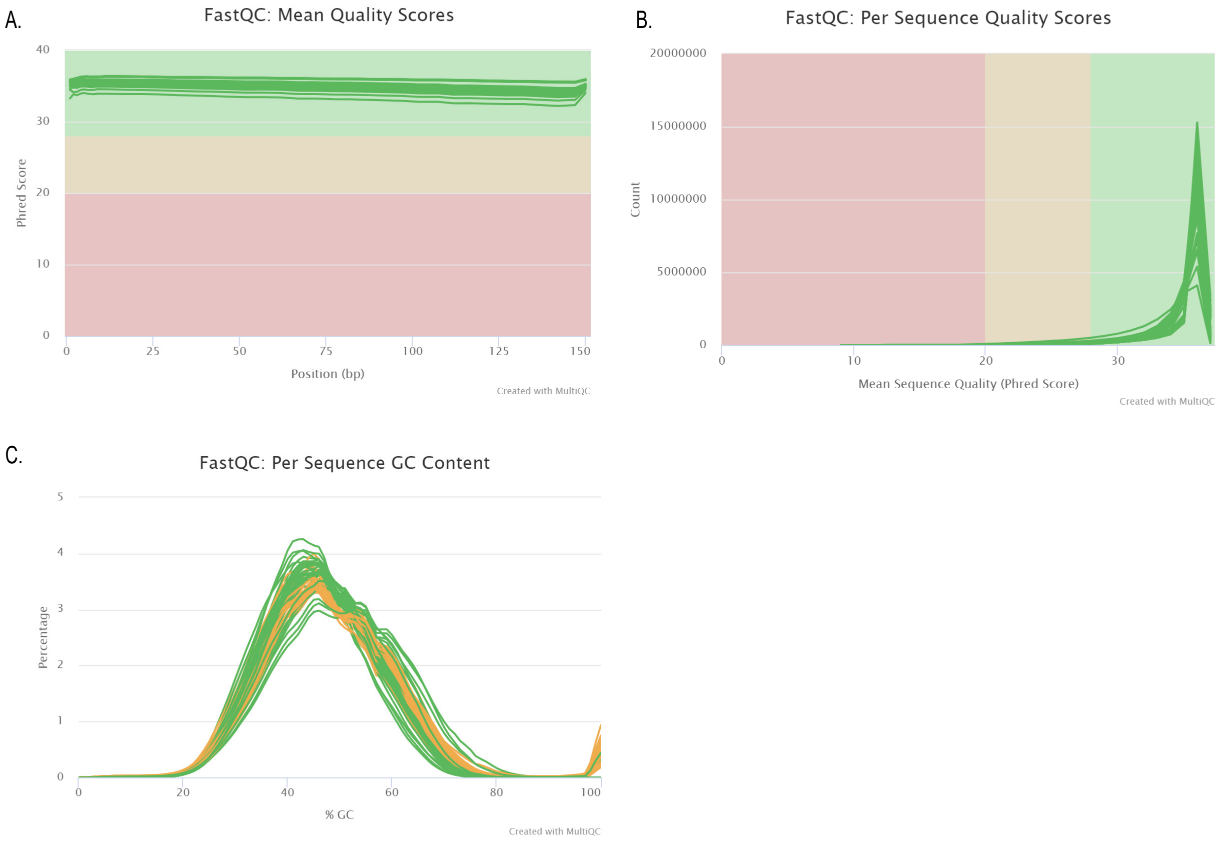

Supplementary Figure 1. Overview of sequence quality utilizing MultiQC.
(A) Per base Phred quality score averaged across each base >33 value in all samples.
(B) Per sequence Phred quality score showing that the majority of subsets of the reads have quality >33.
(C) Per sequence GC content plot indicating roughly normal GC content of all paired end samples.

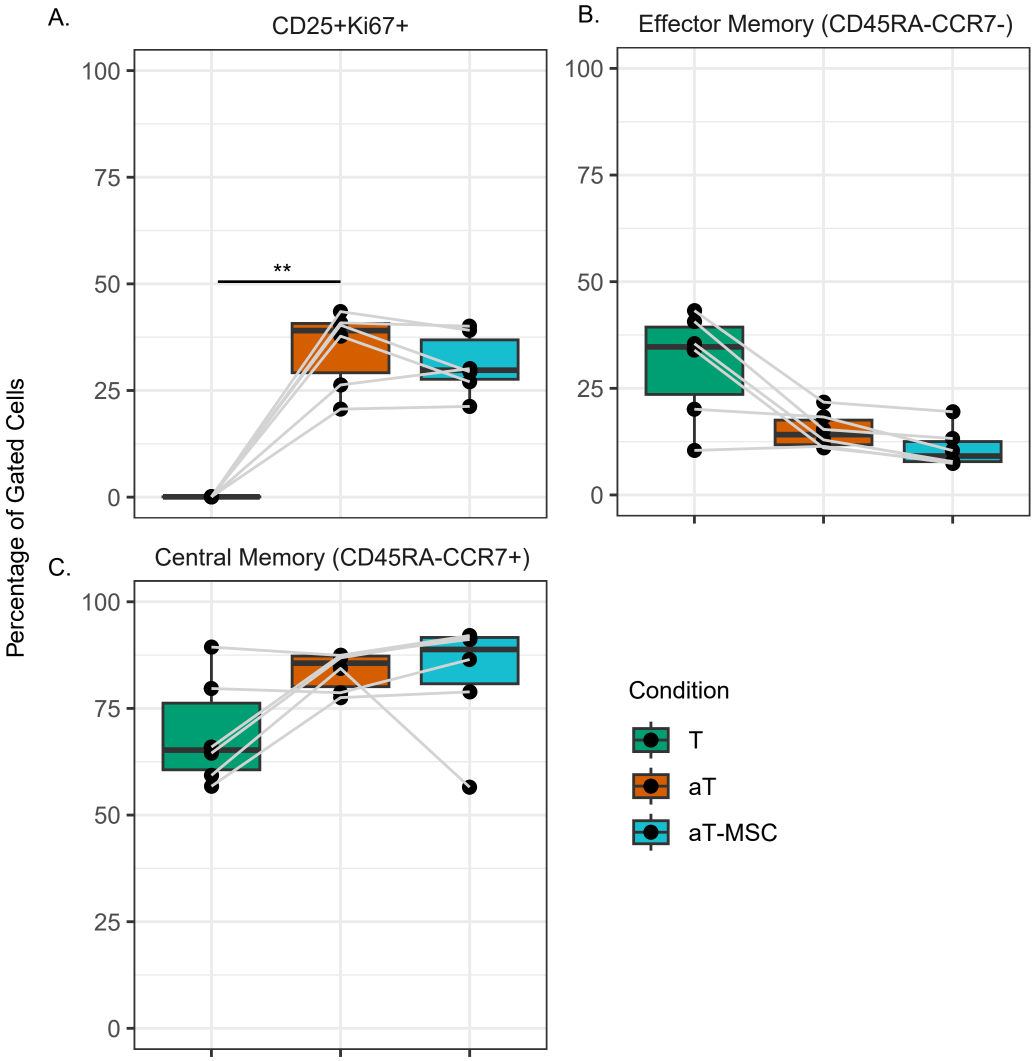

Supplementary Figure 2. Phenotyping of CD4+T cells in MSC co-cultures. (A) CD25+Ki67+ phenotype is used for indication of activated and proliferating CD4+T cells. (B) Effector memory cells, CD45RA-CCR7- and (C) central memory cells CD45RA-CCR7+ were also visualized.

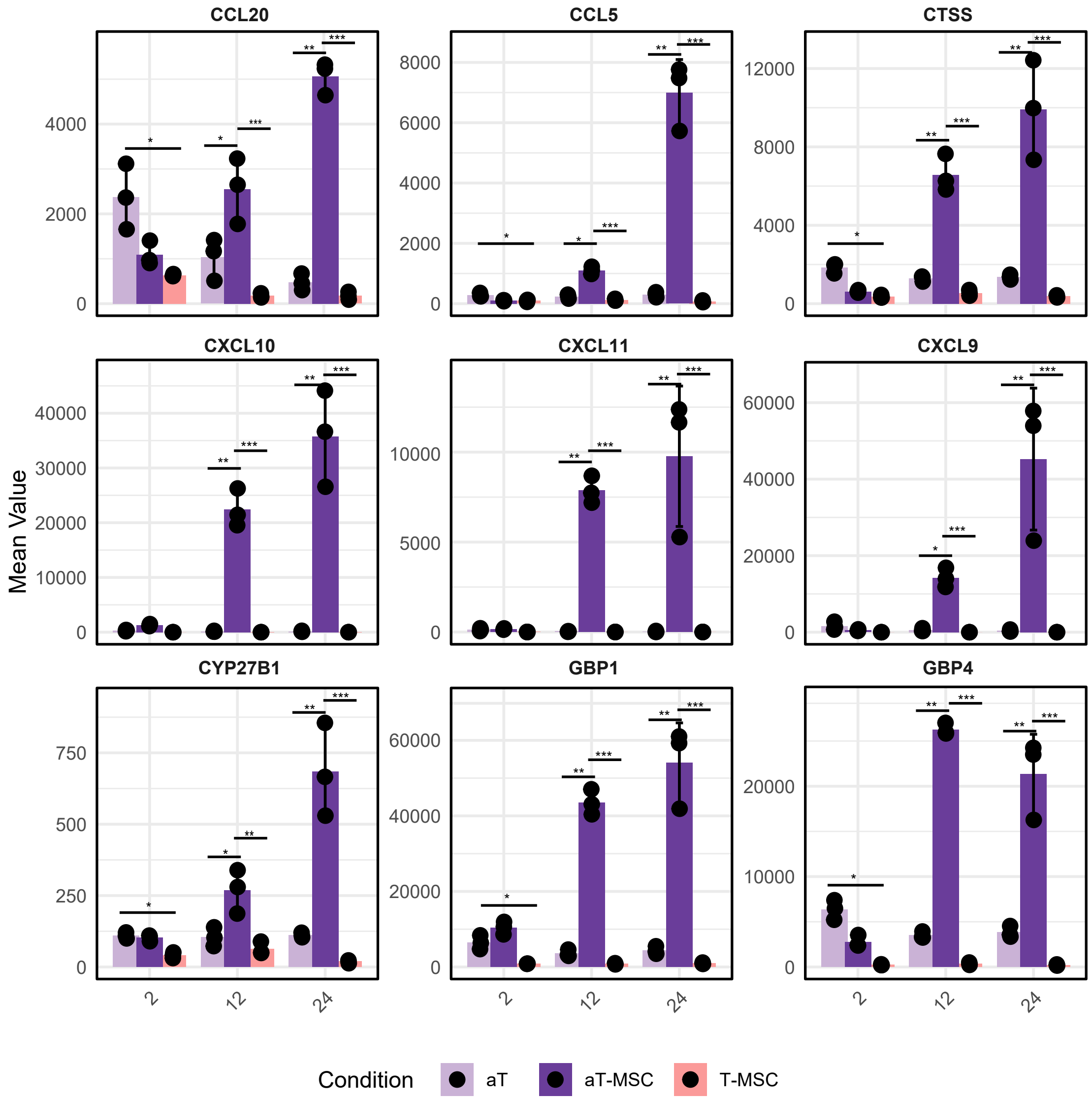

Supplementary Figure 3. Time dependent change of chemotaxis related genes. 9 genes that highly expressed in the presence of MSC were compared between the experimental conditions. Dunn's Multiple Comparison test. *p<0.05, **p<0.01, ***p<0.001, no labelling meaning not significant

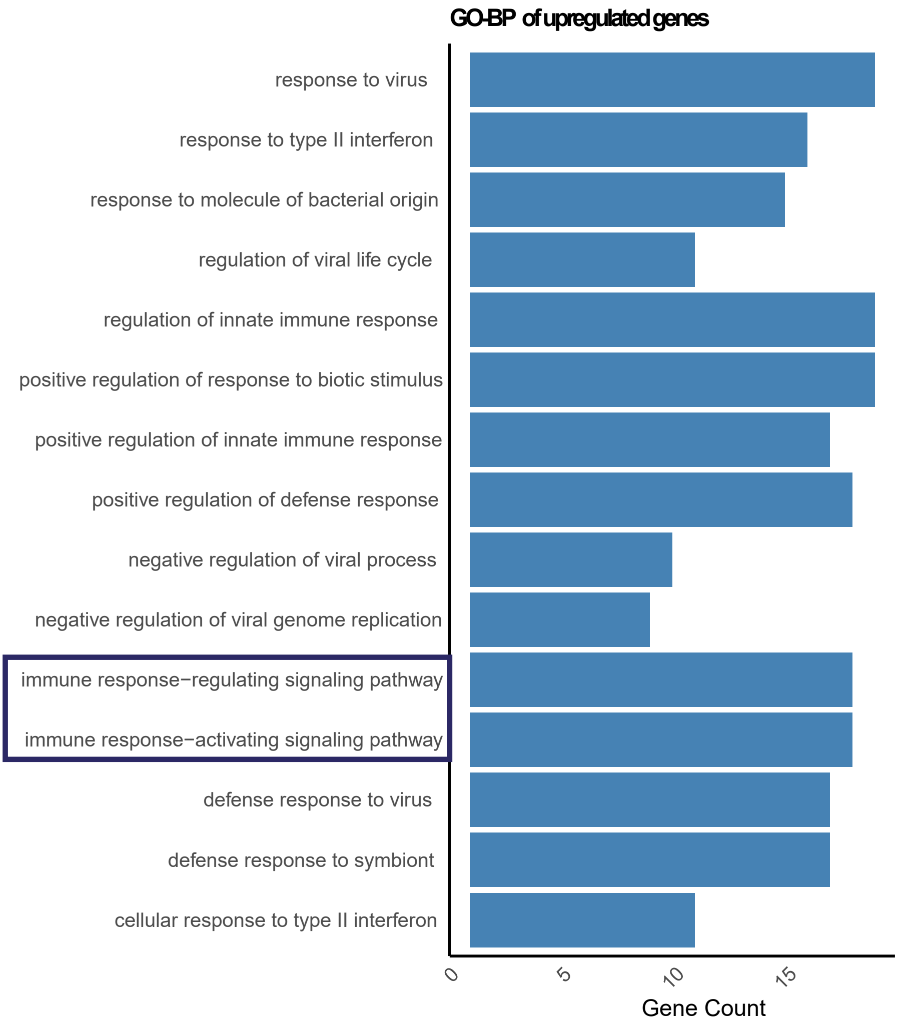

Supplementary Figure 4. GO-BP analysis for all the genes that were upregulated in aT-MSC compared to aT at 24h.
